# SARS-CoV-2 Envelope (E) Protein Binds and Activates TLR2 Pathway: A Novel Molecular Target for COVID-19 Interventions

**DOI:** 10.3390/v14050999

**Published:** 2022-05-08

**Authors:** Rémi Planès, Jean-Baptiste Bert, Sofiane Tairi, Lbachir BenMohamed, Elmostafa Bahraoui

**Affiliations:** 1 Institute of Pharmacology and Structural Biology (IPBS), University of Toulouse, CNRS, 31400 Toulouse, France; remi.planes@ipbs.fr; 2 Institut Toulousain des Maladies Infectieuses et Inflammatoires (INFINITY), INSERM, CNRS, Université Paul Sabatier Toulouse III, 31062 Toulouse, France; jean-baptiste.bert@univ-tlse3.fr (J.-B.B.); sofiane.tairi@inserm.fr (S.T.); 3 Laboratory of Cellular and Molecular Immunology, Gavin Herbert Eye Institute, School of Medicine, University of California Irvine, Irvine, CA 92697, USA; lbenmoha@uci.edu

**Keywords:** SARS-CoV-2, envelop (E) protein, TLR2, CXCL8

## Abstract

This paper presents a molecular characterization of the interaction between the SARS-CoV-2 envelope (E) protein and TLR2. We demonstrated that the E protein, both as a recombinant soluble protein and as a native membrane protein associated with SARS-CoV-2 viral particles, interacts physically with the TLR2 receptor in a specific and dose-dependent manner. Furthermore, we showed that the specific interaction with the TLR2 pathway activates the NF-κB transcription factor and stimulates the production of the CXCL8 inflammatory chemokine. In agreement with the importance of NF-κB in the TLR signaling pathway, we showed that the chemical inhibition of this transcription factor leads to significant inhibition of CXCL8 production, while the blockade of the P38 and ERK1/2 MAP kinases only results in partial CXCL8 inhibition. Overall, our findings propose the envelope (E) protein as a novel molecular target for COVID-19 interventions: either (i) by exploring the therapeutic effect of anti-E blocking/neutralizing antibodies in symptomatic COVID-19 patients, or (ii) as a promising non-spike SARS-CoV-2 antigen candidate for inclusion in the development of next-generation prophylactic vaccines against COVID-19 infection and disease.

## 1. Introduction

SARS-CoV-2, the etiologic agent of the current worldwide COVID-19 pandemic, is a β-coronavirus belonging to the Coronaviridae family. SARS-CoV-2, which emerged in 2019, is the third causative agent of severe acute respiratory syndrome, also named COVID-19 (coronavirus disease 2019). The other two viruses are SARS-CoV and MERS-CoV, which emerged in 2002 and 2012, respectively. SARS-CoV-2 is an enveloped virus with a single-strand positive RNA genome of about 30 kbases and shares 79% nucleotide identity with the genome of SARS-CoV [1]. The virus encodes for four structural proteins, N, M, S and E. While the N protein (46 kDa), in association with the viral RNA, forms the nucleocapsid, the other three structural proteins are located within the viral envelope. The envelope contains three proteins: (i) The “spike” protein (S), a glycoprotein of 180–200 kDa [2], is present as trimers at the surface of the viral particle. It plays a crucial role in the entry of the virus into target cells following its interaction with the ACE2 receptor to induce the fusion of viral and cell target membranes [3]. (ii) The membrane protein (M), a protein of 25–30 kDa, is involved in viral assembly and is the major protein of the envelope. (iii) The envelope (E) protein is an 8.4–12 kDa polypeptide of 76 to 109 amino acids [4,5]. It is a small integral viral membrane protein. Inside infected cells, the E protein is localized in the endoplasmic reticulum, Golgi and ERGIC (ER/Golgi intermediate compartments), where it seems to play an important role in virus assembly and budding [6,7]. In agreement with its role in viral assembly and budding at the endoplasmic reticulum/Golgi compartment where the complete viral particles are produced at the end of the coronavirus life cycle, its mutation or deletion leads to a substantial decrease in the viral replication capacity [8,9]. Thus, the important role of the E protein makes it a potential target for the development of antiviral drug molecules and candidate vaccines [10]. Protein–protein interactions between E and M proteins have been well characterized, as shown by the presence of E–M complexes at the ERGIC level in infected cells [11,12]. It is also interesting to note that the expression of these two proteins is sufficient for the formation of VLP (viral-like particles) [6,13]. By interfering with protein transport via secretory pathways, and with the normal function of the immune system, the E protein could act as a pathogenic factor in the immunopathogenesis associated with SARS-CoV, MERS-CoV and SARS-CoV-2 [8,14]. In fact, in SARS-CoV-infected cells, the E protein anchored to a lipid bilayer is able to adopt a structure forming membrane-integral pores, also named viroporins, with a selective activity for cations including H^+^, K^+^, Na^+^ and Ca^2+^ [15]. Finally, the selective permeability to Ca^2+^ has been reported to be associated with the inflammatory response often observed in ARDS (acute respiratory distress syndrome) [15].

Infection with SARS-CoV-2 is accompanied by deregulation of the control mechanisms of the innate immune response [16,17]. This deregulation is characterized by a delay in IFN-I and III production and also by an exacerbation of the inflammatory response including not only IL-6, TNF-α, IL-1β and IFN-γ but also certain chemokines including CXCL8 [18]. In patients developing critical COVID-19, this dysregulation leads to the establishment of a cytokine storm, a deleterious proapoptotic state in various tissues and organs, including the lungs [19,20,21]. SARS-CoV-2 infection also impacts the adaptive immune response by affecting the normal physiological functions of antigen-presenting cells [22], and also CD4+ and, to a higher degree, CD8+ T cells [23,24].

Understanding the molecular mechanisms responsible, on the one hand, for the control or escape from SARS-CoV-2 detection by innate immune sensors and, on the other hand, for SARS-CoV-2-induced pathological hyper-inflammation is an essential step for the development of effective therapeutic strategies against COVID-19. To achieve this goal, it is important to determine the nature of viral pathogen-associated molecular patterns (PAMPS) and cellular pattern recognition receptors (PRRs) that are engaged in the course of SARS-CoV-2 infection. According to their biochemical and structural characteristics, PRRs are classified into six families, as follows: On the surface of the cell or in endosomes, there are (i) Toll-like receptors (TLRs), (ii) lectin-type C receptors (CLRs), (iii) scavenger receptors and (iv) opsonin receptors. In addition to these transmembrane receptors, cells also express cytosolic and/or nuclear receptors, including: (v) receptors for nucleic acids, RLRs (RIG-I-like receptors), which recognize RNA, and cytosolic DNA sensors called CDSs (including cGAS) and AIM2-like receptors (ALRs, including AIM2); and (vi) NOD-type receptors, NOD-like receptors (NLRs) [25,26]. To date, at least nine PRRs have been reported in the detection of RNA viruses, including TLR7 and 8 (single-stranded RNA), TLR3 (single-stranded RNA), RIG-I and MDA-5 (single- and/or double-stranded RNA, di- or tri-phosphorylated in 5′), DAI/ZBP-1 (RNA with a Z conformation) [27,28] and receptors forming NLRP3 and NLRP1 [29] inflammasomes, as well as helicases of the DDX family, including DDX3 which recognizes the RNA of HIV-1 [30]. More recently, it has been suggested that the envelope (E) protein can be sensed by TLR2 [31]. In addition, the expression of TLR2 and its cofactor MyD88 is associated with enhanced inflammatory responses, particularly in critically ill COVID-19 patients with severe disease [31]. More interestingly, it has been shown, in an ACE2 transgenic mouse model, that blockade of the TLR2 pathway provides protection against the development and lethality of the disease induced by SARS-CoV infection [31].

Considering the important role of innate immune sensors, including TLR2, as potential therapeutic targets in order to alleviate the development of the hyper-inflammation and cytokine storm associated with severe COVID-19, the present study analyzed the interaction between the SARS-CoV-2 envelope (E) protein and human TLR2 at the molecular level. Our findings demonstrate that the SARS-CoV-2 E protein interacted with the TLR2 receptor in a specific and dose-dependent manner, not only in a solid-phase binding assay but also on the cell membrane of TLR2-positive cells, including primary human monocytes and macrophages. Moreover, using HEK-based TLR2 reporter cell lines, we showed that the E protein activates the TLR2 signaling pathway which culminates in the activation of the NF-κB transcription factor and production of inflammatory cytokines/chemokines including CXCL8.

This finding suggests that the envelope (E) protein should be considered a novel target for COVID-19 interventions: either (i) by exploring the therapeutic effect of anti-E blocking/neutralizing antibodies in symptomatic COVID-19 patients or (ii) as a promising non-spike SARS-CoV-2 antigen candidate for inclusion in the development of next-generation prophylactic vaccines against COVID-19 infection and disease.

## 2. Materials and Methods

### 2.1. Ethics Statement

The use of human cells in this study was approved by the Research Ethical Committee of Haute-Garonne, France. Human peripheral blood mononuclear cells (PBMCs) were isolated from buffy coats of healthy human donors. Buffy coats were provided anonymously by the EFS (Etablissement Français du Sang, Toulouse, France). Written informed consent was obtained from the donors under EFS contract N° 21/PVNT/TOU/INSERM01/2011-0059, according to French Decree N° 2007–1220 (articles L1243-4, R1243-61).

### 2.2. Cells

Human embryonic kidney cell lines stably transfected with TLR2 (HEK-TLR2), TLR4 (HEK-TLR4) and HEK-TLR2-blue cells expressing the secreted embryonic alkaline phosphatase (SEAP) reporter genes placed under the control of the IFN-β minimal promoter fused to five NF-κB and AP-1 binding sites and the control HEK cell line (HEK-null) were purchased from InvivoGen. Cells were cultured in DMEM supplemented with 10% FCS, 1% P/S and a selection of antibiotics according to the manufacturer’s instructions (InvivoGen, Toulouse, France).

### 2.3. Virus Inactivation and Purification

SARS-CoV-2 (Occitanie/2020/SCoV2-006) was produced in Vero E6 cells in DMEM medium supplemented with 2% FCS and 1% penicillin/streptomycin. After 3 days of culture at 37 °C, the viral supernatant was harvested, and the virus was inactivated by β-propiolactone treatment overnight at 4 °C as described by Cenac C, et al. [32]. Inactivated viral particles were then pelleted by centrifugation for 2 h at 25,000 rpm as described in [32]. Pellets were then resuspended in PBS, evaluated at 5 mg/mL by the Bradford assay for protein content and stored at −80 °C until use.

### 2.4. Isolation of Human Monocytes

PBMCs were isolated from buffy coats of healthy blood donors (from Etablissement Français du Sang (EFS), Toulouse, France), and monocytes were separated from lymphocytes by positive selection using the magnetic cell sorting technique according to the manufacturer’s instructions (Miltenyi Biotec) and as described in [33].

### 2.5. Generation of Monocyte-Derived Macrophages

To allow the differentiation of monocytes into monocyte-derived macrophages, monocytes were cultured in DMEM medium (Invitrogen) supplemented with 10% fetal calf serum (FCS), 100 IU/mL penicillin, 100 µg/mL streptomycin, 10 ng/mL GM-CSF and 10 ng/mL MCSF. The medium was replaced on day three, and the cells were used for experimentation on day 7 after differentiation into macrophages.

### 2.6. Chemical Products, Proteins and Antibodies

PAM2CSK4, PAM3CSK4, LPS-RS and recombinant nucleocapsid protein fused to His-tag were purchased from InvivoGen. Recombinant soluble E protein from SARS-CoV-2 fused to GST (E-GST) was purchased from Clinisciences. GST, GST-Nef and the corresponding antibodies were produced in our laboratory. Soluble recombinant TLR2 and spike protein fused to His-tag were purchased from R&D systems. Anti-spike rabbit polyclonal antibodies were purchased from Invitrogen. Anti-TLR2 and anti-TLR4 monoclonal antibodies were obtained from eBioscience. Anti-phospho-P65 and anti-total P65 were purchased from Cell Signalling. Bay11-7082, SB202190, PD98059 and RO318220 were purchased from Calbiochem. The MyD88 inhibitor ST 2825 P65 was purchased from MedChemExpress.

### 2.7. Interaction of E Protein with TLR2 in a Solid-Phase Assay

The binding of the recombinant E-GST protein with TLR2 was tested in a solid-phase assay. Briefly, 100 μL of recombinant soluble TLR2 (R&D systems, Minneapolis, MN, USA) at 1 μg/mL was coated for 2 h at room temperature in 96-well plates. After 1 h of saturation with 300 μL of PBS containing 5% non-fat milk and 5 washes with PBS-Tween 0.05%, 100 μL of different concentrations of soluble E-GST protein (1–1000 ng/mL) was added to each well. After 1 h of incubation at 37 °C, 5 washes were performed with PBS-0.05% Tween. Then, the detection of TLR2-E-GST complexes was performed through an additional incubation for 1 h at room temperature with 100 µL of rabbit anti-GST serum previously diluted at 1/500 in PBS-Tween 0.05% containing 5% non-fat milk. After 5 further washes, the TLR2-E-GST-anti-GST complexes were labeled during 1 h incubation at room temperature with 100 µL of anti-rabbit IgG antibodies coupled to horseradish peroxidase (DAKOTA) in PBS-Tween 0.05% containing 5% non-fat milk. After the final 5 washes with PBS-Tween 0.05%, TLR2-E-GST-anti-GST-anti-rabbit-IgG-peroxydase complexes were revealed by the addition of 100 μL of TMB substrate (tetramethylbenzidine). After 15 to 30 min incubation, the peroxidase reaction was stopped with 50 μL of sulfuric acid (2N), and then the optical density was read at 450/570 nm.

### 2.8. Inhibition Assay of E–TLR2 Interaction

The specificity of the E–TLR2 interaction was evaluated in a solid-phase binding assay as described above, except that various amounts of PAM2CSK4 and PAM3CSK4 were added to rTLR2-precoated wells for 1 h before adding a constant amount of soluble E protein (200 ng/mL).

### 2.9. Flow Cytometry Analysis

Monocytes (10^6^) were incubated with GST or GST-E SARS-CoV-2 protein at 0.1–10 µg/mL for 1 h at 37 °C in PBS, BSA 0.5% and NaN_3_ 0.05%. Then, cells were washed 3 times with PBS, BSA 0.5% and NaN_3_ 0.05% to remove unbound proteins. Cells were stained with anti-GST-Alexa 488 (#A-11131, ThermoFisher, Waltham, MA, USA, 1/200) for 1 h at room temperature and washed 3 times with PBS, BSA 0.5% and NaN_3_ 0.05%. Then, cells were fixed with PFA 4%. Data were acquired using a FACSCalibur (BD), Franklin Lakes, NJ, USA.

HEK-TLR2 or HEK-null cells (10^6^) were incubated for 1 h at room temperature (RT) with increasing amounts of β-propiolactone-inactivated SARS-CoV-2 viral particles (0.5–50 µg). After three washes, cell-bound viral particles were detected by immunostaining. Cells were incubated for 1 h at RT with rabbit anti-spike antibodies (Invitrogen #PA5114451) used at 1/500 dilution. After 3 washes, cells were stained, for 30 min at RT, with anti-rabbit IgG produced in donkey coupled to Alexa 488 (Invitrogen #A21206) used at 1/100. Then, cells were washed and fixed with PFA 4%. Data were acquired using a FACSCalibur (BD).

### 2.10. Microscopy Analysis

The analysis of the binding of the E protein to macrophages was analyzed by microscopy. Macrophages (10^6^) were incubated with GST or GST-E SARS-CoV-2 protein at 10 µg/mL for 1 h at 37 °C in PBS, BSA 0.5% and NaN_3_ 0.05%. Then, cells were washed 3 times with PBS, BSA 0.5% and NaN_3_ 0.05% to remove unbound proteins. Afterwards, cells were washed 3 times with PBS, stained with Hoechst and anti-GST-Alexa 488 (1/500) for 1 h at room temperature and washed 3 times with PBS, BSA 0.5% and NaN_3_ 0.05%. Finally, cells were fixed with PFA 4% before imaging. Images were acquired using an EVOS M700 (Invitrogen) at 40× magnification.

In parallel experiments, we evaluated the capacity of inactivated SARS-CoV-2 viral particles to bind HEK cells expressing TLR2 (HEK-TLR2) or not (HEK-null). To this end, 10^6^ HEK-TLR2 or HEK-null cells were preincubated with β-propiolactone-inactivated virus for 1 h at RT, in PBS, BSA 0.5%, NaN_3_ 0.05% and human AB serum 4%. After 3 washes with 500 µL of PBS, BSA 0.5% and NaN_3_ 0.05%, cell-bound viral particles were detected by immunostaining. Cells were incubated for 1 h at room temperature with rabbit anti-spike antibodies used at 1/500 dilution, washed 3 times and further incubated, for 30 min at RT, with anti-rabbit IgG produced in donkey coupled to Alexa 488 used at 1/100. Then, cells were washed and fixed with PFA 4%. Images were acquired using an EVOS M700 (Invitrogen) at 40× magnification.

### 2.11. Cell-Based Biological Assays

Primary human monocytes or macrophages cells (10^6^ cells) or HEK-null, HEK-TLR2 or HEK-TLR4 cell lines (2.5 × 10^5^ cells) were plated in 24-well plates and treated with the E protein, or PAM3CSK4 and PAM2CSK2 as positive controls at the indicated concentrations. Untreated cells were used as negative controls. To block TLR2, anti-TLR2 was added in the cell culture medium 1 h before treatment with the E protein. To inhibit cell signaling pathways, cells were incubated with chemical inhibitors 30 min before treatment with the E protein. To inhibit the binding of the E protein to cell membrane TLR2, the E protein (used at 200 ng/mL) was preincubated with rTLR2 (20 ng/mL) for 1 h at RT, before being added to HEK-TLR2 cells. Cell supernatants were collected 18 h after E treatment and frozen at −20 °C before further analysis.

### 2.12. Phosphorylation Analysis of NF-kB P65 Subunit and Western Blot Analysis

HEK-TLR2 cells (2.5 × 10^5^ cells) were treated for 30 or 60 min with the E protein (1 µg/mL), or with GST (1 µg/mL) or PAM3CSK4 (10 ng/mL) as negative and positive controls, respectively. Then, cells were lysed and prepared for immunoblotting as previously described [34].

To investigate the capacity of inactivated SARS-CoV-2 viral particles to bind HEK cells expressing TLR2 (HEK-TLR2) or not (HEK-null), cells were incubated for 1 h at room temperature, with an increasing amount of inactivated virus (0.1–100 µg) in PBS, BSA 0.5% and NaN_3_ 0.05%. After 3 washes with PBS, cells were lysed in RIPA buffer, and samples were prepared for immunoblotting as described previously [34]. Anti-spike antibodies (1/1000) were used to detect TLR2-bound viral particles. Anti-GAPDH was used as a loading control.

### 2.13. NF-kB Assay Using HEK-Blue and HEK-TLR2-Blue

The capacity of the E protein or the inactivated SARS-CoV-2 virus to activate NF-kB was tested using HEK-TLR2-blue cells (InvivoGen). In this assay, HEK-TLR2-blue cells stably transfected with the SEAP (secreted embryonic alkaline phosphatase) gene under the control of the NF-κB promoter were plated at 5 × 10^4^ cells per well in 96-well plates one day before the experiment. The following day, cells were treated with the E protein (1–1000 ng/mL), or inactivated SARS-CoV-2 virus (0.025–25 µg), in 100 µL of cell culture medium at the indicated concentration. Eighteen hours after treatment, supernatants were collected, and quantification of SEAP was performed according to the manufacturer’s instructions (InvivoGen).

### 2.14. CXCL8 Quantification by ELISA

Cells were stimulated with various amounts of the E protein (1–1000 ng/mL) or inactivated SARS-CoV-2 in the presence or absence of anti-TLR2 antibodies (Anti-hTLR2-IgA, InvivoGen #6120-43-02) or IgA control antibodies (InvivoGen, #6437-43-02). After 18 h of stimulation at 37 °C, supernatants were harvested and stocked at −20 °C until CXCL8 quantification by ELISA according to the instructions of the manufacturer (R&D system).

### 2.15. Statistical Analyses

Statistical analysis was performed using GraphPad Prism software v.5. All results are expressed as means +/− SDs. All experiments were performed a minimum of three times. Differences in the means for the different groups were tested using one-way ANOVA followed by the Bonferroni post hoc test. A *p*-value < 0.05 was considered statistically significant. Statistical significance comparing different groups is denoted with * for *p* < 0.05, ** for *p* < 0.01 and *** for *p* < 0.001, while ns means non-significant.

## 3. Results

### 3.1. SARS-CoV-2 Envelope (E) Protein Interacts Directly and Physically with TLR2

**Figure 1:**
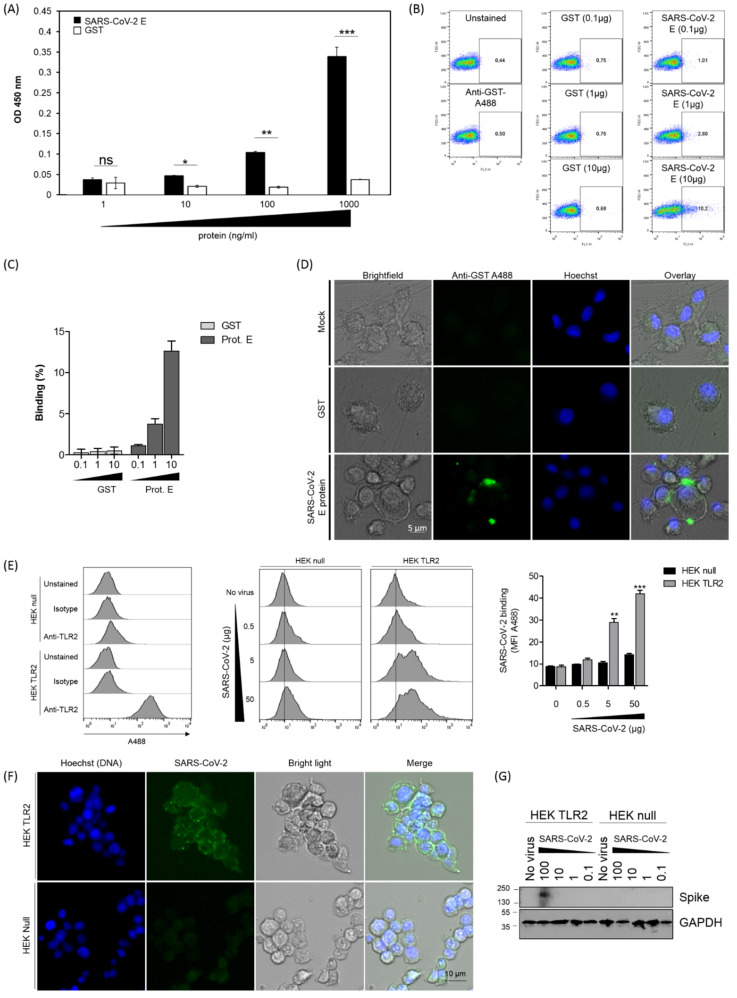
Binding of SARS-CoV-2 protein to human TLR2**.** (**A**) Soluble recombinant human TLR2 (100 µL at 1 µg/mL) was coated in 96-well plates. After saturation, various amounts of the E-GST protein (1 ng/mL–1000 ng/mL) were added for 2 h at 37 °C. TLR2 E-GST complexes were revealed by a solution of anti-GST sera follow by anti-anti-GST conjugated to HRP. (**B**) Primary human monocytes were incubated with 0.1 to 10 µg/mL of GST or GST-E SARS-CoV-2 protein. Cells were stained with anti-GST (1/1000). Data were acquired using a FACScalibur. One representative experiment is shown. (**C**) Quantification of the SARS-CoV-2 E protein or GST control binding to human monocytes from 3 different experiments acquired using a FACScalibur. (**D**) Primary human macrophages were incubated with 10 µg/mL of GST or GST-E SARS-CoV-2 protein. Cells were stained with anti-GST (1/500). Images were acquired using an EVOS M700 microscope. (**E**) HEK-TLR2 or HEK-null (10^6^) cells were preincubated for 1 h with increasing amounts of inactivated SARS-CoV-2 (0.5–50 µg). The staining of SARS-CoV-2 complexes was performed with rabbit anti-spike antibodies used at 1/500 and donkey anti-rabbit IgG complexed with Alexa 488 used at 20 µg/mL. (**F**) HEK-TLR2 or HEK-null cells previously plated in 24-well plates were incubated for 1 h with various amounts of inactivated SARS-CoV-2 at RT. After 3 washes, binding of SARS-CoV-2 to HEK cells was evaluated by microscopy, and bound viral particles were detected by anti-spike antibodies. (**G**) HEK-TLR2 or HEK-null cells previously plated in 24-well plates were incubated for 1 h with various amounts of inactivated SARS-CoV-2 (0.1–100 µg) at RT. After washing and cell lysis, cell-associated viral particles were evaluated after SDS-PAGE and Western blotting using anti-spike antibodies. Statistical significance comparing different groups is denoted with * for *p* < 0.05, ** for *p* < 0.01 and *** for *p* < 0.001, while ns means non-significant.

In order to analyze the capacity of the SARS-CoV-2 envelope (E) protein to interact with TLR2 at a molecular level, a solid-phase assay was used to test the binding of various amounts of E protein (1–1000 ng/mL) to a constant amount of precoated human recombinant TLR2 (1 μg/mL). The results show that the E protein bound to TLR2 in a dose-dependent manner (Figure 1A). In contrast, no significant binding to TLR2 was observed when the experiment was performed with GST instead of the E protein (Figure 1A). It was further observed that the E protein, but not GST used as a control, was also able to bind to human primary monocytes (analysis by flow cytometry, Figure 1B,C) and to human primary macrophages (analysis by microscopy, Figure 1D).

In addition, to demonstrate that the native, virus-associated E membrane protein was able to bind to TLR2, the binding of SARS-CoV-2 viral particles to HEK-TLR2 cell lines was evaluated using three complementary approaches. In the first approach, the binding of SARS-CoV-2 to TLR2 was evaluated by flow cytometry. The results shown in Figure 1E indicate that the SARS-CoV-2 viral particles bound to HEK-TLR2 in a dose–response manner, while no significant binding was observed with HEK-null cells. In the second approach, the binding of SARS-CoV-2 to TLR2 was evaluated by microscopy. Staining with anti-spike antibodies demonstrated a clear binding of SARS-CoV-2 viral particles to HEK-TLR2 but not to HEK-null cell lines (Figure 1F). In the third approach, SARS-CoV-2 viral particles (0.1–100 µg/mL) were incubated with HEK-TLR2 or HEK-null cells, and the bound viral particles were evaluated, after cell lysis, by SDS-PAGE and Western blotting analysis. In these conditions, a specific band with strong staining, having an electrophoretic mobility compatible with the expected molecular mass (180–200 kDa) of the spike protein, was detected with the stimulation performed with the highest dose of the virus (Figure 1G). In contrast, no band corresponding to the spike protein was detected when the experiments were performed with the HEK-null cell line, thus confirming the specificity of the SARS-CoV-2–TLR2 interaction (Figure 1G).

Altogether, these results demonstrate that the SARS-CoV-2 envelope (E) protein, as a native protein expressed on viral particles, interacted specifically and in a dose-dependent manner with the human soluble recombinant TLR2 and also with TLR2 expressed at the surface of primary human monocytes, macrophages and a HEK-TLR2 engineered cell line.

### 3.2. PAM2CSK4 and PAM3CSK4 Antagonize SARS-CoV-2 E Protein Binding to TLR2

**Figure 2:**
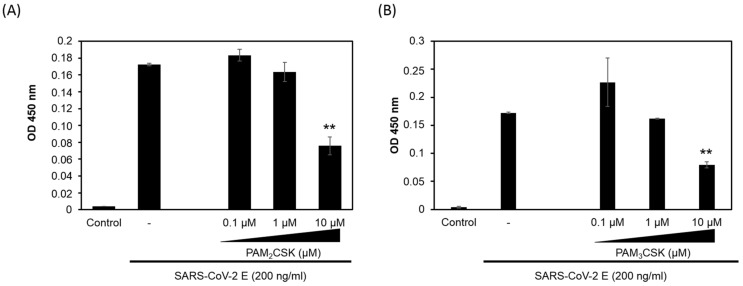
PAM2CSK4 and PAM3CSK4 interfere with SARS-CoV-2 E binding to TLR2. The specificity of E–TLR2 interaction was evaluated by testing the capacity of TLR2 ligands (**A**) PAM2CSK4 (0.1–10 µM) and (**B**) PAM3CSK4 (0.1–10 µM) to inhibit this interaction. Statistical significance comparing different groups is denoted with ** for *p* < 0.01.

PAM2CSK4, a synthetic diacylated lipopeptide ligand of TLR2/TLR6, and PAM3CSK4, a synthetic triacylated lipopeptide ligand of TLR2/TLR1, have been identified as specific ligands of TLR2 [35,36,37,38]. Thus, in order to characterize the specificity of the interaction of the E protein with TLR2, we evaluated the capacity of these two ligands, PAM2CSK4 and PAM3CSK4, to inhibit E–TLR2 interaction. The experiment was performed as described in Figure 1, using a constant concentration of the E protein (200 ng) but in the presence of escalating amounts of PAM2CSK4 or PAM3CSK4 (0.1 µM to 10 µM). Both ligands inhibited E–TLR2 interaction in a dose-dependent manner (Figure 2A,B). However, only partial inhibition, albeit exceeding 50%, was obtained with PAM2CSK4 and PAM3CSK4 used at 10 µM. Thus, these characterizations demonstrate that E protein–TLR2 interaction is specific, as demonstrated by the capacity of PAM2CSK4 and PAM3CSK4 to inhibit this interaction.

### 3.3. SARS-CoV-2 E Protein and Inactivated SARS-CoV-2 Stimulate the Production of CXCL8 Inflammatory Chemokine by Recruiting TLR2 Pathway

**Figure 3:**
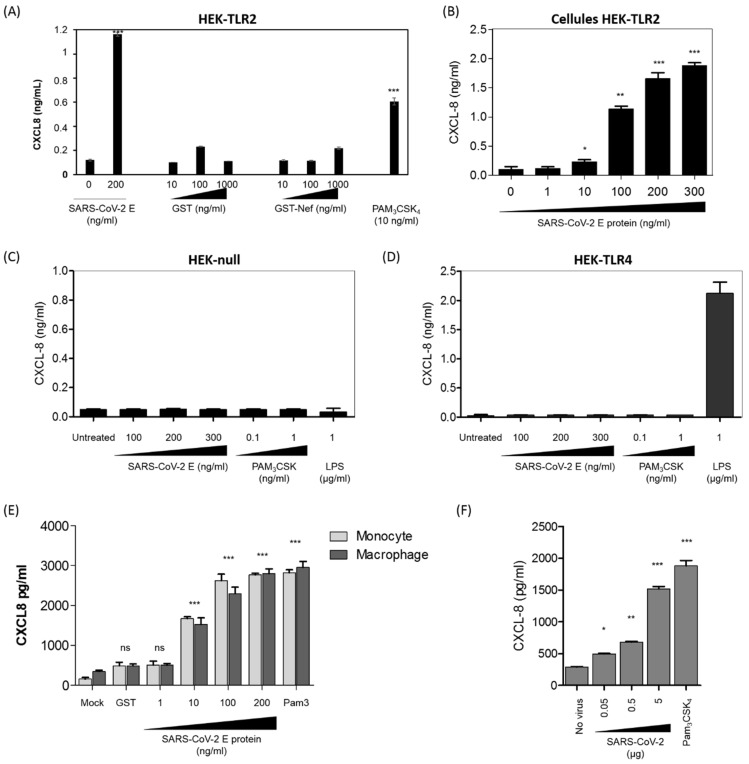
The E protein stimulates the production of the CXCL8 chemokine in a TLR2-dependent manner. (**A**) The HEK-TLR2 cell line was stimulated with the E protein (200 ng/mL), GST (10–1000 ng/mL), GST-Nef (10–1000 ng/mL) or PAM3CSK4 (10 ng/mL). CXCL8 chemokine production in the cell supernatants was quantified by ELISA. (**B**) Production of CXCL8 in cell supernatants of HEK-TLR2 cells stimulated by escalating concentrations of the E protein (1–300 ng/mL). (**C**,**D**) Production of CXCL8 in cell supernatants of HEK-null (**C**) and HEK-TLR4 (**D**) cell lines stimulated with the E protein (1–100 ng/mL) or with PAM3CSK4. (**E**) Primary human monocytes and macrophages were stimulated with the E protein (1–200 ng/mL). Stimulation with GST (200 ng/mL) or PAM3CSK4 (1000 ng/mL) was used as a negative and a positive control, respectively. After 20 h of treatment, the cell supernatant was collected and CXCL8 chemokine production in the cell supernatants was quantified by ELISA. (**F**) Primary human monocytes (10^6^) were stimulated with an increasing amount (0.05 µg/mL–5 µg/mL—equivalent viral proteins present in the purified viral pellet) of β-propiolactone-inactivated SARS-CoV-2. Stimulation with PAM3CSK4 was used as a positive control. After 20 h of treatment, the cell supernatant was collected and CXCL8 chemokine production in the cell supernatants was quantified by ELISA. Statistical significance comparing different groups is denoted with * for *p* < 0.05, ** for *p* < 0.01 and *** for *p* < 0.001, while ns means non-significant.

In order to study the biological consequences of E–TLR2 interaction, we tested the capacity of the E protein to stimulate the production of CXCL8 in a HEK cell line-based assay using cells stably transfected with the human TLR2 (HEK-TLR2) or TLR4 (HEK-TLR4) receptors or HEK-null (transfected with an empty plasmid). As previously shown by our group, activation of the TLR-dependent pathway in HEK cell lines stimulates the production of measurable amounts of the CXCL8 chemokine, while other TLR-dependent cytokines including TNF-α, IL-6 and IL-10 are barely detectable. Consequently, the production of CXCL8 by HEK cell lines was used as a marker of the TLR response [39]. The results presented in Figure 3A show that the E protein from SARS-CoV-2 (200 ng/mL) stimulated the production of CXCL8 in TLR2-expressing HEK cell lines, while GST and GST-Nef, two unrelated SARS-CoV-2 gene products, used as controls, did not stimulate significant production of CXCL8 when used at concentrations up to 1 µg/mL (Figure 3A). In additional controls, no production of CXCL8 was obtained in the supernatants of the unstimulated HEK-TLR2 cell line, while a clear production of CXCL8 was noted following stimulation by the synthetic ligand of TLR2 PAM3CSK4 (Figure 3A). The specificity of the activation of the TLR2 pathway by the E protein was further demonstrated by showing that the E protein induced the production of CXCL8 in a dose-dependent manner, with the lowest amount of E protein that gave a detectable CXCL8 production being around 10 ng/mL (Figure 3B). The specificity of E–TLR2 pathway activation was further supported by the fact that no CXCL8 production was obtained in the HEK-null (Figure 3C) or HEK-TLR4 (Figure 3D) cell lines. The latter control also demonstrated the absence of endotoxins as contaminants in our recombinant E protein, as demonstrated by the absence of any activation of the TLR4 pathway (Figure 3C,D).

Taking into account the data obtained with the HEK-TLR2 cell line, we tested the capacity of the E protein to activate the production of CXCL8 in human primary monocytes and macrophages. In this test, human monocytes and macrophages were stimulated by increasing concentrations of the E protein (1 ng/mL to 200 ng/mL) for 20 h, and CXCL8 was quantified by ELISA as described above. The results obtained show that the E protein stimulated the production of CXCL8 in both primary human monocytes and macrophages (Figure 3E).

In order to demonstrate that the viral-associated E protein, present in the viral particle, is also able to stimulate the TLR2 pathway in human monocytes, we showed that β-propiolactone-inactivated SARS-CoV-2 viral particles were able to stimulate the production of the CXCL8 chemokine in a dose-dependent manner (Figure 3F).

Altogether, our results show that the SARS-CoV-2 E protein, by recruiting the TLR2 pathway at least, stimulated the production of CXCL-8.

### 3.4. The Stimulation of CXCL8 Production by SARS-CoV-2 E Protein or SARS-CoV-2 Viral Particles Is Reversed by Soluble rTLR2 and Anti-TLR2 Antibodies

**Figure 4:**
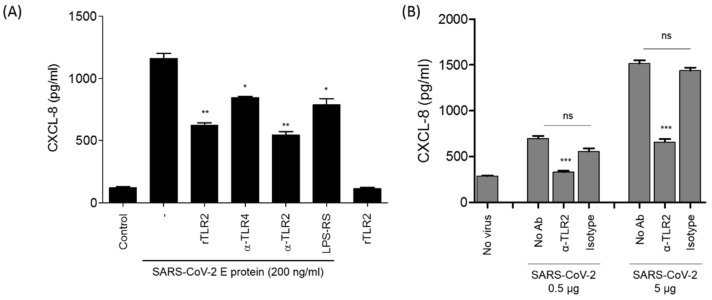
Inhibition of E-induced CXCL8 production by soluble recombinant TLR2 or by anti-TLR2 antibodies. (**A**) The HEK-TLR2 cell line was stimulated with the E protein (200 ng/mL) in the presence or absence of recombinant TLR2 (20 ng/mL), anti-TLR2 and anti-TLR4 antibodies or LPS-RS (10 µg/mL), and recombinant TLR2 (20 ng/mL) alone was used as a control. (**B**) HEK-TLR2 cells were stimulated with inactivated SARS-CoV-2 (0.5–5 µg/mL) in the presence of anti-TLR2 antibodies or their isotypes (10 µg/mL). After 20 h of treatment, the cell supernatant was collected and CXCL8 chemokine production in the cell supernatants was quantified by ELISA. Statistical significance comparing different groups is denoted with * for *p* < 0.05, ** for *p* < 0.01 and *** for *p* < 0.001, while ns means non-significant.

The specificity of the recruitment of the TLR2 pathway by the E protein was further characterized in complementary assays using either soluble recombinant TLR2 (rTLR2) or anti-TLR2 blocking antibodies. The results show that incubation of rTLR2 with the E protein before stimulation of HEK-TLR2 cells inhibited the capacity of the E protein to stimulate the TLR2 response by about 50%, as measured by the production of CXCL8. Importantly, no significant CXCL8 production was obtained with rTLR2 alone (Figure 4A). Interestingly, this result also indicates the absence of endotoxins in the preparation of the rTLR2 used. In agreement with the effect of rTLR2, we showed that anti-TLR2 antibodies, used at 5 µg/mL, inhibited E-induced CXCL8 production by about 60% (Figure 4A), while only moderate inhibition, of less than 30%, was observed when anti-TLR4 antibodies (5 µg/mL) were used as isotype controls. We also tested the effect of LPS-RS, a specific antagonist of TLR4. Used at 10 µg/mL, LPS-RS led to modest inhibition of E2-induced CXCL8 production, by about 18% (Figure 4A). These moderate inhibitions may have resulted from the steric hindrances caused by the presence of anti-TLR4 antibodies and LPS-RS.

The specificity of E protein–TLR2 interaction was further supported by the capacity of anti-TLR2 antibodies to partially inhibit SARS-CoV-2-induced CXCL8, compared to that of isotype control antibodies (Figure 4B). This partial inhibition of CXCL8 in the presence of anti-TLR2 antibodies suggests the implication of other SARS-CoV-2 PAMPs, in addition to the E protein, in the production of CXCL8.

Altogether, these results confirm the recruitment of the TLR2 pathway by the E protein as demonstrated by the capacity of soluble recombinant TLR2 and anti-TLR2 antibodies to strongly block CXCL8 chemokine production in HEK-TLR2 and human monocyte cells stimulated by the SARS-CoV-2 E protein or inactivated SARS-CoV-2 viral particles.

### 3.5. SARS-CoV-2 E Protein and Inactivated SARS-CoV-2 Activate NF-kB as a Signature of the Recruitment of TLR2 Pathway

**Figure 5:**
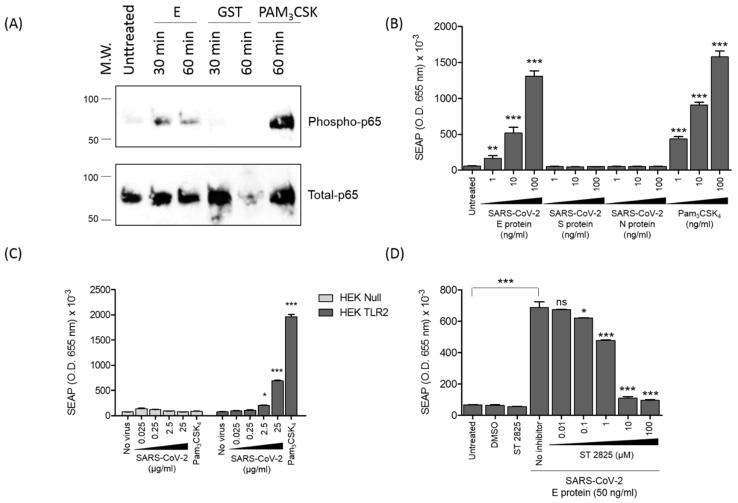
The E protein stimulates the activation of NF-kB. (**A**) HEK-TLR2 cells were stimulated with the E protein, GST or PAM3CSK4 for 30 or 60 min. Phosphorylation of P65 was analyzed by SDS-PAGE and Western blot by using specific anti-phospho-P65 (**upper panel**) or anti-total p65 antibodies (**lower panel**). (**B**) The HEK-TLR2 cell line, stably transfected with SEAP (secreted embryonic alkaline phosphatase), was treated with escalating concentrations of the envelope, spike or nucleocapsid protein or with PAM3CSK4 for 18 h, and SEAP activity was quantified in the cell supernatants. (**C**) HEK-blue TLR2 or HEK-blue null cells were stimulated with inactivated SARS-CoV-2 (0.025–25 µg) and analyzed at 45 min as described in Figure 1B. (**D**) The HEK-TLR2 cell line was treated with the envelope protein in the presence of an increasing amount of the MyD88 inhibitor ST 2825 for 18 h, and SEAP activity was quantified in the cell supernatants. Statistical significance comparing different groups is denoted with * for *p* < 0.05, ** for *p* < 0.01 and *** for *p* < 0.001, while ns means non-significant.

Activation of all TLR pathways leads to activation of NF-κB. In order to be activated, NF-κB must be phosphorylated on its subunits p65 and p50, but also on its inhibitor subunit IkB, thus leading, on the one hand, to the nuclear translocation of p65 into the nucleus, where it binds on NF-κB sites at the CXCL8 promotor element sequence, and, on the other hand, to the dissociation, ubiquitination and proteasomal degradation of IkB [40]. In the present study, the effect of the E protein on the activation of NF-κB was evaluated by monitoring its effect on the phosphorylation of the p65 subunit. For this purpose, HEK-TLR2 cells were stimulated for 30 or 60 min with the E protein (1 µg/mL) or with GST or PAM3CSK4 as negative and positive controls, respectively. Both at 30 and 60 min post-stimulation, the E protein led to the phosphorylation of p65 (Figure 5A, lanes 3 and 4). Only slight phosphorylation of p65 was observed in unstimulated cells (Figure 5A, lane 2) and in cells stimulated with the GST protein (Figure 5, lanes 5 and 6). As expected, strong phosphorylation was obtained following stimulation with PAM3CSK4 (Figure 5A, lane 7).

Then, the effect of the E protein on the activation of NF-κB was further characterized in a more functional assay, based on the evaluation of the capacity of the E protein or inactivated SARS-CoV-2 viral particles to activate the expression of the gene product of the soluble SEAP protein placed under the control of the NF-κB inducible promotor. For this, the HEK-TLR2 cell line stably transfected with the SEAP gene under the control of NF-κB was stimulated with various amounts of the E protein (1–100 ng/mL) or inactivated SARS-CoV-2 viral particles (0.025–25 µg) for 18 h. The expression of the enzymatic activity of the soluble SEAP protein was then measured in the cell supernatants after 30 min of reaction. The results reported in Figure 5B clearly show the presence of SEAP enzymatic activity in the cell supernatants of HEK-blue TLR2 cells treated with the SARS-CoV-2 envelope protein or the positive control PAM3SCK4, but not in those treated with the spike or nucleocapsid protein. In a negative control, no significant SEAP enzymatic activity was observed in supernatants of unstimulated HEK-TLR2 cells (Figure 5B).

In order to demonstrate that the native viral E protein was also able to activate the NF-kB pathway, HEK-TLR2 and HEK-null cells, stably transfected with the SEAP gene under the control of NF-kB, were stimulated, using the same protocol as described above, with increasing concentrations of inactivated SARS-CoV-2 (0.025–25 µg). The results obtained show a dose- and time-dependent response in HEK-TLR2, while no significant responses were observed in HEK-null cells stimulated with the same amounts of the virus (Figure 5C).

To investigate the implication of the MyD88 pathway in the activation of NF-κB downstream of E–TLR2 interaction, we evaluated the capacity of the MyD88 inhibitor (ST 2825) to interfere with the activation of the SEAP reporter gene following E protein treatment in the HEK-blue TLR2 model. The results show that the MyD88 inhibitor (ST 2825) inhibited the activation of the SEAP reporter gene following SARS-CoV-2 E treatment in a dose-dependent manner (Figure 5D).

Altogether, these results show that the SARS-CoV-2 E protein and SARS-CoV-2 viral particles are able to recruit and engage the TLR2 pathway, leading to activation of the transcription factor NF-kB, as demonstrated by the phosphorylation of the p65 NF-κB subunit and the transactivation of the SEAP gene under the control of the NF-κB promotor site.

### 3.6. SARS-CoV-2 E Protein Activation of CXCL8 Production Is Dependent on NF-kB Pathway

**Figure 6:**
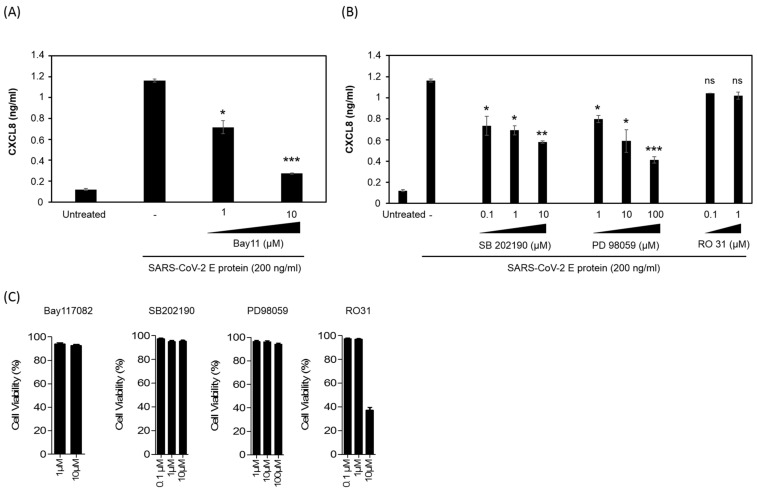
Inhibition of E-induced CXCL8 production by an NF-kB inhibitor but not by P38 and ERK1/2 MAP kinase and PKC inhibitors. (**A**) Inhibition of the E-induced CXCL8 chemokine in the presence of an NF-kB inhibitor. HEK-TLR2 cells were stimulated with the E protein (200 ng/mL) in the presence of the chemical inhibitor of NF-kB, Bay11, used at 1 and 10 µM. Production of CXCL8 in cell supernatants was quantified by ELISA. (**B**) HEK-TLR2 cells were pretreated with P38 MAP kinase inhibitor SB202190 (0.1–10 µM), ERK1/2 MAP kinase inhibitor PD 98059 (1–100 µM) or PKC inhibitor RO318220 (0.1–10 µM) for 1 h, before stimulation with the E protein (200 ng/mL). Produced CXCL8 in cell supernatants was quantified by ELISA. (**C**) The cytotoxicity of the used chemical inhibitors: SB202190, PD98059 and RO318220, was tested in a trypan blue exclusion assay at the used concentrations. Statistical significance comparing different groups is denoted with * for *p* < 0.05, ** for *p* < 0.01 and *** for *p* < 0.001, while ns means non-significant.

To evaluate the role of NF-kB in the control of CXCL8 production in response to E stimulation in HEK-TLR2, cells of the HEK-TLR2 cell line were pretreated for 60 min with various non-toxic concentrations (1–10 μM) of the NF-kB inhibitor Bay11-7082 before stimulation with the E protein (200 ng/mL) (Figure 6C). After 18 h of culture, CXCL8 production was quantified in cell supernatants. Dose-dependent inhibition of CXCL8 production was observed in the presence of Bay11-7082, demonstrating the crucial role of the transcription factor NF-kB in the control of the gene expression of the CXCL8 chemokine (Figure 6A).

MAP kinases, including P38 MAPK and ERK1/2 MAPK, have been reported to participate in the activation of AP1 and CREB and thus, indirectly via AP1 and CREB, contribute to the increased expression of the CXCL8 gene product. Considering these contributions, we tested the effect of the inhibition of P38 MAPK and ERK1/2 MAPK on the production of CXCL8 following activation of the HEK-TLR2 cell line by the E protein. For this test, HEK-TLR2 cells were pretreated for 60 min with non-toxic concentrations of SB202190 (0.1–10 µM) and PD98059 (1–100 µM) as inhibitors of P38 MAPK and ERK1/2 MAPK, respectively, before treatment with the E protein at 200 ng/mL (Figure 6C). Both inhibitors produced a partial inhibitory effect, reaching 55% and 70% of inhibition by SB202190 and PD98059, respectively, when used at the highest concentrations (Figure 6B).

Because P38 and ERK1/2 are activated downstream of PKCs, a large family of serine/threonine kinases, we also evaluated the effect of PKCs on the production of CXCL8 by E-stimulated HEK-TLR2 cells. Cells were pretreated with various concentrations of R0318220, an inhibitor of all PKC isoforms, before stimulation with the E protein at 200 ng/mL and quantification of CXCL8 in cell supernatants as described above. No evident inhibition was observed in the presence of the PKC inhibitor Ro318220 used at 0.1 and 1 µM (Figure 6B). However, the apparent inhibition observed at 10 µM of the inhibitor was further related to the cytotoxic effect of the 10 µM concentration of RO318220 as evaluated by the cytotoxicity assay measuring LDH release, a signature of cell death, or in the trypan blue exclusion assay (Figure 6C).

Taken together, our results demonstrate the direct physical interaction between the E protein of SARS-CoV-2 and TLR2. This interaction engages the activation of the TLR2 pathway, leading to the activation of the transcription factor NF-κB, which, in contrast to the ERK1/2 and P38 MAP kinases, seems to play a major role in the production of the CXCL8 chemokine.

## 4. Discussion

Recent work by Zheng and colleagues provided genetic evidence that the TLR2 pathway contributes to overwhelming production of inflammatory cytokines (particularly TNF-α, IL-6 and IFN-γ) during infection by SARS-CoV-2 and other β-coronaviruses, following recognition of the E protein [31]. In light of this recent finding, the present study provides further characterizations of E–TLR2 interaction. Specifically, the results demonstrate that the SARS-CoV-2 E protein interacts physically in a dose-dependent manner with the soluble recombinant TLR2 receptor and also with cell membrane TLR2 of primary human monocytes and macrophages. Additionally, our findings show that the E protein from SARS-CoV-2 activates the TLR2 and MyD88 pathways, leading to activation of the transcription factor NF-κB, which seems to play a major role in the production of the CXCL8 chemokine, in contrast to the ERK1/2 and P38 MAP kinases, whose inhibition results in only partial inhibition of CXCL8.

TLR2 was originally described as recognizing ligands of bacterial origins [35,36,37,38] that include diacyl and triacylglycerol moieties, proteins and polysaccharides. However, it is currently assumed that recognition of TLR2 is not limited to bacterial ligands but concerns a broader set of molecules, including viral proteins (review in [41]). These TLR2 viral ligands include glycoprotein B of Cytomegalovirus, the hepatitis C core and NS3 proteins and hemagglutinin (H) of measles virus [41]. Thus, the E protein from SARS-CoV-2 extends the list of viral TLR2 ligands. The diversity of molecules recognized by the TLR2 receptor may be explained by its capacity to form heterodimers with TLR1, TLR6 or TLR10 and to benefit from the help of additional cofactors including CD14 and CD36 [41]. However, the involvement of TLR2 in the interaction with the E protein raises a number of questions. Crystallographic studies of the complex between TLR2/TLR1 and its triacylated lipopeptide ligand PAM3CSK have allowed the sites of interaction between TLR2/TLR1 and its ligand PAM3CSK to be determined [42]. The structures of the lipopeptide TLR2 ligands PAM3CSK and PAM2CSK contain three and two lipid chains, respectively. By interacting with the hydrophobic pocket of TLR2, these lipid chains allow heterodimerization of TLR2/TLR1 by PAM3CSK and TLR2/TLR6 by PAM2CSK, and the recruitment of downstream adapters including Mal/MyD88, thus allowing the activation of the TLR2 pathway [42]. It is therefore important to question how the E protein of SARS-CoV-2, which does not have a lipid tail, can interact with and activate the TLR2 pathway. Analysis of the primary structure of the E protein reveals two hydrophilic regions in the N- and C-terminal parts of the molecule, separated by a large hydrophobic domain, which could present an affinity for the hydrophobic pocket of TLR2. In addition, it has been reported that the E protein also exists in the form of homo-oligomeric multimers [15,43,44] which, by interacting with the hydrophobic pockets of TLR2, TLR1 and TLR6, could bridge the formation of heterodimers of TLR2/TLR1 or TLR2/TLR6, and even of homodimers of TLR2/TLR2. Further structural research studies are needed to confirm these hypotheses. Our data showing that the PAM3CSK and PAM2CSK synthetic ligands interfere with E–TLR2 binding suggest that the E protein and PAM2CSK/PAM3CSK bind TLR2 on partially overlapping sites.

In this study, we demonstrated a direct physical binding of the E protein to TLR2 in a solid-phase binding assay. However, this assay does not provide information about the functionality of this interaction, nor does it indicate whether it induced structural rearrangements or oligomerizations of TLR2. However, our findings showing that the E protein is also able to bind to cell membrane TLR2 of primary monocytes and macrophages, to activate the transcription factor NF-κB and to stimulate the production of the CXCL8 chemokine represent strong arguments in favor of the capability of the E protein to recruit and engage the TLR2 pathway. NF-κB is an important transcription factor that participates in the control of the expression of cytokine genes that are involved in the immune and inflammatory responses [40,45,46].

Our findings show that, in addition to the soluble recombinant E protein, the membrane-associated viral E protein present in inactivated SARS-CoV-2 is also able to bind and activate the TLR2 pathway, in a dose-dependent manner. The results suggest that the recombinant E protein conserves a biological activity similar to that of its native membrane-associated form. However, we cannot exclude the possibility that some of the signals obtained with the entire SARS-CoV-2 viral particles (binding, NF-κB activation and CXCL8 production) may also be, at least partially, related to other viral proteins and factors.

Activation of TLR2 highlights a diverse number of intracellular signaling pathways that culminate in the transcription of several immunity-related genes including pro-inflammatory cytokines and chemokines that play an important role in shaping the innate and adaptive immune responses, as well as in tissue homeostasis. Our data show that the E protein activates NF-κB and demonstrate that this activation is essential for CXCL8 production in the HEK-TLR2 cell line. The partial inhibition obtained in the presence of the P38 and ERK1/2 MAP kinase inhibitors is in line with the secondary role of these pathways in the activation of the AP1 and CREB transcription factors in CXCL8 gene expression [47,48]. In addition to NF-κB, the promotor element sequence of the CXCL8 gene also contains binding sites for additional transcription factors, including AP-1 (activating protein), CREB (cAMP response element binding protein), C/EBP (CAAT/enhancer-binding protein), CHOP (C/EBP homologous protein) [49] and C/EBP beta (also named NF-IL-6) [48]. While NF-κB is crucial for the expression of the CXCL8 gene, other transcription factors, such as AP1 and CREB, seem to play a secondary role by acting on the stability of mRNA and through synergistic action with NF-κB on the expression of the CXCL8 gene, thus contributing to an efficient production of the CXCL8 gene product [47,48].

Activation of the TLR pathway by viruses could have either a beneficial or a harmful effect on the host [50,51]. For example, the use of an animal model elegantly demonstrates that TLR7-dependent type I interferon production by plasmacytoid dendritic cells (pDCs) confers protection against mouse hepatitis virus (MHV) viral infection [52]. Similarly, type I interferon production has also been observed following pDC interaction with SARS-CoV [52] and SARS-CoV-2 [53]. Accordingly, in order to escape from the TLR-mediated immunity, viruses have developed several strategies to interfere with signal transduction downstream of TLR pathways [51,54]. Dysregulated activation of the TLR pathway has been associated with enhanced pathogenesis. This is the case for the TLR4 pathway, which is involved in pathogenesis of IAV, EBOV and DENV infections, while treatment with TLR4 antagonists (Eritoran) reduces cytokine/chemokine production and alleviates disease symptoms [55]. Other viruses take advantage of the TLR pathway for their own benefit. Our group and others have shown that HIV-1, through its Tat protein, activates the TLR4 pathway, leading to the upregulation of several immunosuppressive factors including IL-10, PD-L1 and IDO-1 [33,34,56,57]. This is also the case for measles virus, which subverts the TLR2 pathway by its hemagglutinin (H) protein in order to upregulate the expression of its own entry receptor, CD150 [58]. In the case of SARS-CoV-2, data from Zheng and colleagues suggest that the TLR2 pathway is involved in disease pathogenesis rather than viral control [31].

Although the exact pathway of COVID-19 pathogenesis is still unknown, recent data have demonstrated that elevated levels of pro-inflammatory cytokines in serum, including CXCL8, are associated with enhanced disease pathogenesis and mortality. Accordingly, inflammatory mediators are promising therapeutic targets to alleviate COVID-19 pathogenesis [20,59,60,61]. Thus, understanding the molecular determinants responsible for inflammatory cytokine production in the course of SARS-CoV-2 infection could provide future therapeutic targets. Several SARS-CoV-2 components have been described as triggering inflammatory cytokine production, including the detection of viral RNA by MDA-5 [62], TLR8 [63] and TLR7 [52,53], activation of ACE2 by the spike (S) protein in epithelial cells [64] and activation of TLR2 by the E protein [31]. However, the relative contribution of each pathway in immune protection or pathogenesis warrants further studies. It should be noted that the work of Zheng, et al. showed that, unlike the E protein, the S protein does not seem to induce a significant inflammatory reaction [31]. Our results show that the spike and nucleocapsid proteins of SARS-CoV-2 are not able to activate the TLR2 pathway in contrast to the envelope protein. This difference underlines the importance of considering the E protein as a therapeutic target. Our findings show that the E protein induces CXCL8 production in a TLR2-, MyD88- and NF-κB-dependent manner when tested in the HEK-TLR2 cell line model. Thus, this model provides an important tool that could be used to screen antagonist compounds that could be used as therapeutic drugs. The production of CXCL8, a known neutrophil chemoattractant, is consistent with reports describing a high number of circulating neutrophils and associated injury in the airway and lung tissues of COVID-19 patients [65,66]. Regarding the pathological deleterious effect of CXCL8 in COVID-19 patients, we could consider targeting the E protein for therapeutic purposes, either by immunotherapy approaches by administering neutralizing anti-E antibodies to COVID-19 patients in intensive care units (ICUs), or by a vaccine approach, by combining the E protein with an immunogen in future candidate vaccines against COVID-19.

## 5. Conclusions

We demonstrated that the SARS-CoV-2 envelope protein binds and activates the human TLR2 pathway. E protein–TLR2 interaction leads to the downstream activation of the NF-κB transcription factor that stimulates the production of CXCL8. These data support a role for the envelope protein of SARS-CoV-2 in the elevated levels of pro-inflammatory cytokines associated with COVID-19 pathogenesis. Overall, our findings propose the envelope (E) protein as a novel molecular target for COVID-19 interventions.

## Data Availability

Data supporting the reported results can be provided by E.B.
